# Retinoic acid promotes myogenesis in myoblasts by antagonizing transforming growth factor-beta signaling via C/EBPβ

**DOI:** 10.1186/s13395-015-0032-z

**Published:** 2015-03-18

**Authors:** Émilie Lamarche, Neena Lala-Tabbert, Angelo Gunanayagam, Catherine St-Louis, Nadine Wiper-Bergeron

**Affiliations:** Graduate Program in Cellular and Molecular Medicine, Faculty of Medicine, University of Ottawa, Ottawa, Ontario Canada; Department of Cellular and Molecular Medicine, Faculty of Medicine, University of Ottawa, Ottawa, Ontario Canada

**Keywords:** Retinoic acid, C/EBPβ, TGFβ-signaling, Skeletal muscle

## Abstract

**Background:**

The effects of transforming growth factor-beta (TGFβ) are mediated by the transcription factors Smad2 and Smad3. During adult skeletal myogenesis, TGFβ signaling inhibits the differentiation of myoblasts, and this can be reversed by treatment with retinoic acid (RA). In mesenchymal stem cells and preadipocytes, RA treatment can function in a non-classical manner by stimulating the expression of Smad3. Smad3 can bind to and prevent the bzip transcription factor CCAAT/enhancer-binding protein beta (C/EBPβ) from binding DNA response elements in target promoters, thereby affecting cell differentiation. In skeletal muscle, C/EBPβ is highly expressed in satellite cells and myoblasts and is downregulated during differentiation. Persistent expression of C/EBPβ in myoblasts inhibits their differentiation.

**Methods:**

Using both C2C12 myoblasts and primary myoblasts, we examined the regulation of C/EBPβ expression and activity following treatment with TGFβ and RA.

**Results:**

We demonstrate that treatment with RA upregulates Smad3, but not Smad2 expression in myoblasts, and can partially rescue the block of differentiation induced by TGFβ. RA treatment reduces C/EBPβ occupancy of the Pax7 and Smad2 promoters and decreased their expression. RA also inhibits the TGFβ-mediated phosphorylation of Smad2, which may also contribute to its pro-myogenic activities. TGFβ treatment of C2C12 myoblasts stimulates C/EBPβ expression, which in turn can stimulate Pax7 and Smad2 expression, and inhibits myogenesis. Loss of C/EBPβ expression in myoblasts partially restores differentiation in the presence of TGFβ.

**Conclusions:**

TGFβ acts, at least in part, to inhibit myogenesis by upregulating the expression of C/EBPβ, as treatment with RA or loss of C/EBPβ can partially rescue differentiation in TGFβ-treated cells. This work identifies a pro-myogenic role for Smad3, through the inhibition of C/EBPβ’s actions in myoblasts, and reveals mechanisms of crosstalk between RA and TGFβ signaling pathways.

**Electronic supplementary material:**

The online version of this article (doi:10.1186/s13395-015-0032-z) contains supplementary material, which is available to authorized users.

## Background

The canonical transforming growth factor-beta (TGFβ) pathway involves the binding of dimerized TGFβ family ligands to a constitutively active TGFβ type II cell surface receptor [1]. Ligand binding by the type II receptor promotes its association with the TGFβ type I receptor that is both phosphorylated and activated by the type II receptor [2]. The activated type I receptor then phosphorylates receptor Smad transcription factors (Smad2 and Smad3), which bind to Smad4 and translocate to the nucleus to regulate gene transcription [3,4]. Activation and consequent nuclear translocation of Smad2 and Smad3 by the TGFβ type 1 receptor is achieved by phosphorylation of distinct serine residues (Ser465/467 for Smad2 and Ser423/425 for Smad3) in their respective C-terminal tails [5-7].

While much is known about the activation of Smad3 activity, very little is known about the transcriptional pathways regulating Smad3 expression and the functional consequences of this activation in different cell systems. Smad3 expression is inhibited by isoprenoids in a Sp1/Sp3-dependent fashion [8], and mitogen-activated protein kinase A (MAPK) activity can stimulate Smad3 expression, by inhibiting Sp1 binding to a region between −849 and −408 of the Smad3 promoter [9]. Treatment with retinoic acid (RA) has also been shown to upregulate Smad3 mRNA expression in T cells, mesenchymal stem cells, and preadipocytes [10,11].

During myogenesis, treatment with TGFβ is known to potently, yet reversibly, inhibit the differentiation process [12-19]. TGFβ signaling can inhibit myogenesis through activation of Smad3, which can interfere with the formation of muscle regulatory factor-containing transcriptional complexes [12,13]. The muscle phenotype has been investigated in a Smad3 knockout model, in which a premature stop was introduced in exon 7, producing a truncated Smad3 lacking the C-terminal 89 amino acids encoded by exon 8, which contains the serine residues phosphorylated by the activated TGFβ type I receptor [18,20,21]. Loss of the C-terminal domain crippled TGFβ-mediated responses. Interestingly, Smad3 knockout mice had fewer satellite cells, the muscle stem cell primarily responsible for regeneration and repair; reduced myoblast proliferation; and smaller fiber caliber [20]. Moreover, muscle regeneration after acute injury was also reduced in the Smad3 knockout mice [21]. These findings suggested that Smad3 assumes a pro-myogenic role in muscle precursors, in sharp contrast to the potently anti-myogenic role of TGFβ signaling.

While both Smad2 and Smad3 bind the same DNA response element in target promoters and are both activated by the same receptor, there is growing evidence that these factors are not redundant in function. In the kidney, where TGFβ induces tubulo-interstitial fibrosis, Smad3 and Smad2 have been shown to regulate a different subset of genes [22,23]. In pancreatic ductal adenocarcinoma, Smad3 and Smad2 exert opposite effects on growth and cellular migration, and in primed pluripotent cells, Smad3 was shown to be dispensable for the maintenance of the undifferentiated state [24,25]. Indeed, TGFβ has even been shown to inhibit the expression of Smad3 [26] further suggesting that Smad3 can counteract Smad2 activities in some systems.

In mesenchymal stem cells and preadipocytes, treatment with RA has been shown to influence the differentiation of adipocytes and osteoblasts at least in part by stimulating the expression of Smad3. In these systems, Smad3 acts in a non-classical manner, in that it is not C-terminally phosphorylated, but nonetheless affects the expression of CCAAT/enhancer-binding protein alpha (C/EBPα) and Runx2, the master regulators of adipogenesis and osteoblastogenesis, respectively [11,18,27-30]. Both C/EBPα and Runx2 are transcriptional targets of C/EBPβ, a bzip transcription factor involved in numerous cellular differentiation processes. C/EBPβ interacts with the Smad3 MH1 domain, which blocks C/EBPβ’s ability to bind its DNA response elements in target promoters. As such, increased Smad3 levels can abrogate C/EBPβ-mediated transcriptional responses [11]. During adipogenesis, RA treatment and the consequent increase in Smad3 expression prevents interaction of C/EBPβ with the C/EBPα promoter, decreasing expression of the master regulator resulting in the inhibition of differentiation. In contrast, RA can promote osteoblastogenesis by preventing C/EBPβ from interacting with the Runx2 promoter, where it acts as a negative regulator [27,29].

In muscles, C/EBPβ is expressed in satellite cells where it acts to maintain the undifferentiated state. Induction of satellite cells to differentiate is accompanied by a decrease in C/EBPβ expression; loss of satellite cell marker Pax7 expression, a C/EBPβ target gene; and the induction of myogenic regulatory factors such as myogenin [31]. Herein, we identify C/EBPβ as a novel TGFβ target gene that mediates, at least in part, the anti-myogenic effects of this signaling pathway. We demonstrate that C/EBPβ expression is upregulated by TGFβ treatment, and this leads to an increase in C/EBPβ target gene expression. Further, we identify Smad2 as a novel C/EBPβ target, creating an anti-myogenic feed-forward control loop. Loss of C/EBPβ expression, or interference with its ability to bind DNA through treatment with RA, can rescue myogenesis in TGFβ-treated cells.

## Methods

### Western analysis

Protein extracts were harvested with protease and phosphatase inhibitors (Santa Cruz Biotechnology, Santa Cruz, CA, USA) and were analyzed using the following antibodies: C/EBPβ (C-19, Santa Cruz Biotechnology), Smad2/3 (Cell Signaling Technology, Danvers, MA, USA), myogenin, Pax7, and myosin heavy chain (MF-20) primary antibodies from DSHB (Iowa City, IA, USA); and phospho Smad2/3 (Abcam, Cambridge, U.K.). β-Actin (Sigma-Aldrich, St-Louis, MO, USA) was used as a loading control. HRP-conjugated secondary antibodies were from GE Healthcare (Buckinghamshire, U.K.). Chemiluminescence images were captured using the Luminescent Image Analyzer LAS-4000 (Fujifilm Life Science, Tokyo, Japan), and quantifications were done using ImageJ (U.S. National Institutes of Health, Bethesda, MD, USA, http://imagej.nih.gov/ij/, 1997–2014).

### Cell culture

C2C12 myoblasts (ATCC, Manassas, VA, USA) were cultured and maintained in Dulbecco’s modified Eagle’s medium (DMEM; Wisent, Saint-Bruno, QC, Canada) containing 4.5 g/L glucose, 110 mg/L sodium pyruvate, and 584 mg/L l-glutamine and supplemented with 10% heat-inactivated fetal bovine serum (FBS; Invitrogen, Carlsbad, CA, USA). To stimulate skeletal muscle differentiation, 80% confluent C2C12 cells were treated with DMEM containing 1% heat-inactivated horse serum (HS; Invitrogen) for 4 days. 3T3L1 cells (ATCC) were maintained in DMEM containing 4.5 g/L glucose, 110 mg/L sodium pyruvate, and 584 mg/L l-glutamine and supplemented with 10% heat-inactivated calf serum (HI-CS, Invitrogen). Primary myoblasts were freshly isolated from wild-type and C/EBPβ conditional knockout mice as previously described [31]. Briefly, a tamoxifen-sensitive CreER DNA recombinase driven by the *Pax7* locus was used to excise *Cebpb* from homozygous mice bearing Loxp sites inserted on both ends of the *Cebpb* coding sequence. Excision of *Cebpb* was achieved with a 48 h treatment of isolated primary myoblasts with 2 μM 4-OH tamoxifen (Sigma-Aldrich). Isolated primary myoblasts were cultured in DMEM supplemented with 1% penicillin and streptomycin (Wisent) containing 20% FBS, 10% HS, 10 ng/ml FGF (Peprotech, Rocky Hill, NJ, USA), and 2 ng/ml HGF (Peprotech). Culture media and growth factors were replenished every day. Upon reaching confluence, primary myoblasts were switched to DMEM containing 2% FBS and 10% HS (low serum conditions) for 2 days in the presence of TGFβ or vehicle. Media and TGFβ were replenished every day.

### Retroviral infection

Replication-incompetent pLXSN-based retroviruses (Clontech, Palo Alto, CA, USA) were generated in Phoenix Ampho packaging cells (ATCC) and have been described previously [30]. Virus was harvested 48 h after transfection. Following infection of C2C12 myoblasts, cells were selected in growth medium supplemented with 400 ug/mL G418 for 7 days to generate pooled stable cell lines.

### Reagents

All-*trans* retinoic acid (Sigma-Aldrich) was used at a concentration of 1 nM. TGFβ1 (R&D Systems, Minneapolis, MN, USA) was used at a concentration of 1 ng/ml for C2C12 cells and 5 ng/ml for primary myoblasts and changed every 24 h for the duration of the treatment.

### RT-qPCR

For quantitative reverse transcription polymerase chain reaction (RT-qPCR), total RNA was extracted using the RNeasy Mini Kit (Qiagen, Hilden, Germany) and contaminating DNA was digested with DNase (Ambion, Life Technologies, Austin, TX, USA). Subsequently, RNA was reverse transcribed using the iScript kit (Bio-Rad, Hercules, CA, USA) according to manufacturer’s instructions. Real-time PCR reactions were performed with iTaq SYBR Green (Bio-Rad) on a Mx3005p thermocycler (Stratagene, La Jolla, CA, USA). Relative fold induction was determined using the ΔΔCt method [32] following normalization with 18S rRNA.

### Differentiation and fusion indices

To assess differentiation of C2C12 cells and primary myoblasts, immunohistochemistry was performed using anti-myosin heavy chain antibody (MF-20) from DHSB, which recognizes the fast and slow sarcomeric myosin heavy chains. The antibody was incubated overnight, followed by a Cy3-conjugated donkey anti-mouse IgG secondary antibody (Jackson ImmunoResearch, West Grove, PA, USA) for 1 h. DAPI (0.5 μg/ml) counterstain was used to label chromatin. Pictures were taken of a minimum of six random field of view at × 10 magnification per well. The differentiation index is defined as the number of myosin heavy chain positive nuclei divided by the total number of nuclei. The fusion index is described as the number of myosin heavy chain positive nuclei in myotubes divided by the total number of myotubes.

### Chromatin immunoprecipitation assay

C2C12 myoblasts were cultured in growth or differentiation conditions and treated with RA or vehicle for 24 h as indicated. Chromatin immunoprecipitation (ChIP) analysis was performed as described [30] using antibodies for C/EBPβ (C-19; Santa Cruz Biotechnology), RAR (M-454; Santa Cruz Biotechnology), or non-immunized normal rabbit IgG (Invitrogen) as control, incubating at 4°C overnight. After sonication, DNA fragments were purified using the QIAquick PCR Purification Kit (Qiagen) and amplified by qPCR. Primer sequences for the Pax7 promoter were as follows: forward 5′-CCCGAACTGGCCCCCTTTCC-3′ and reverse 5′-TCCCCCGGAGGACTGGAACG-3′. Primer sequences for the intronic RARE region of the Smad3 promoter were as follows: forward 5′-ATGACTTGTTCCTGTCCTTC-3′ and reverse 5′-GCTAGGCAGAGTTCCCAGAA-3′. Primer sequences for the Smad2 promoter were as follows: forward 5′-AAGTCCCTGGAGGGAATGGA-3′ and reverse 5′-CACTGTAGGCAGAGCAGGTT-3′.

### Statistical analysis

Statistical analysis was performed using GraphPad Prism software (GraphPad Software, La Jolla, CA, USA, www.graphpad.com). The student’s *t* test was used when comparing a control and experimental condition in one group. One-way ANOVA followed by Tukey’s post-hoc test was used when comparing one factor in three or more groups. Two-way ANOVA followed by Bonferroni’s post-hoc test was used when comparing two factors in control and experimental conditions. Post-hoc tests followed only statistically significant ANOVA results (*p* < 0.05). Where multiple comparisons are possible, means are marked with a letter code. Means with different letters are significantly different from one another, meeting a minimum cutoff of *P* < 0.05. All experiments are representative of a minimum of three biological replicates, as indicated. Data represents the mean; error bars represent the SEM.

## Results

### RA regulates Smad3 expression in myoblasts

**Figure 1:**
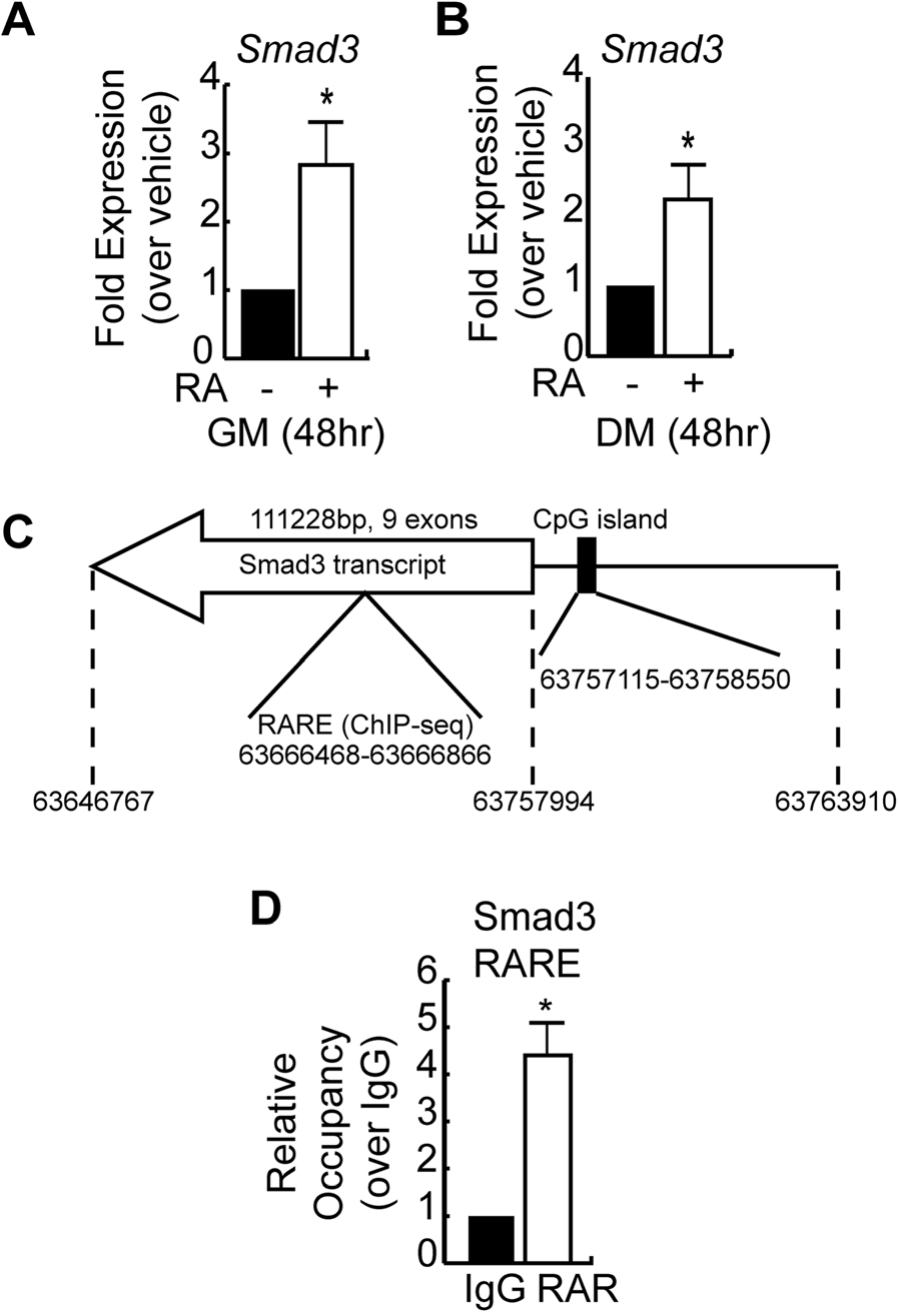
**Retinoic acid upregulates Smad3 expression in myoblasts. (A)** RT-qPCR analysis of *Smad3* mRNA expression in C2C12 myoblasts cultured in growth medium and treated with vehicle or RA for 48 h. Data is shown as fold expression over vehicle-treated condition. Error bars are the SEM, *P < 0.05, *n* = 3. **(B)** RT-qPCR analysis of *Smad3* mRNA expression in C2C12 myoblasts cultured in differentiation medium and treated with vehicle or RA for 48 h. Data is shown as fold expression over vehicle-treated condition. Error bars are the SEM; *P < 0.05, *n* = 3. **(C)** Schematic representation of the mouse *Smad3* locus found on the minus strand of chromosome 9 using the Mouse Dec. 2011 (GRCm38/mm10) Assembly. Location of the transcript, including 5′ and 3′ UTRs, is indicated as well as the predicted CpG island (%CG = 65.9%, length = 1,436 bp, ObsCpG/ExpCpG =0.80). The position of a RARE identified by ChIP-seq analysis in ES cells undergoing neurogenesis (GSM482750) between exon 3 and 4 of the Smad3 gene is also indicated. **(D)** Analysis of retinoic acid receptor (RAR) occupancy of the RARE in the intronic region of the mouse *Smad3* gene by chromatin immunoprecipitation and qPCR in C2C12 cells in growth medium. Data represents the mean; error bars are the SEM; *P < 0.05, *n* = 3.

Consistent with our observations in mesenchymal stem cells and preadipocytes, both proliferating myoblasts cultured in growth medium (GM) and myoblasts cultured in low serum conditions to induce differentiation (differentiation medium, DM) had increased *Smad3* mRNA expression following treatment with 1 nM RA (Figure 1A,B) [11,27]. Treatment with RA resulted in a significant 3-fold increase in *Smad3* mRNA expression in growth medium as compared to vehicle-treated controls (Figure 1A). Similarly, under differentiation conditions, a 2-fold increase in *Smad3* mRNA was observed as compared to vehicle-treated controls (Figure 1B). It should be noted that 1 nM RA did not adversely affect cell growth or differentiation, while higher concentrations of RA were toxic to the cells (Additional file 1: Figure S1).

*In silico* analysis of the mouse *Smad3* gene and the upstream 5-kb region of the predicted promoter revealed no canonical retinoic acid response elements (RARE) by which RA could induce transcription by binding to the retinoic acid receptor: retinoid X receptor (RAR:RXR) heterodimer. Indeed, the promoters driving expression of *Smad3* in mouse, rat, and humans are quite divergent, with the exception of large CpG islands (Figure 1C). These CG-rich regions are prone to methylation, and it has previously been demonstrated that the co-Smad Smad4 is silenced through this mechanism [33]. Methylation of the Smad3 promoter has also been demonstrated in humans [34]. Despite this, incorporation of 5-azacytidine (AZA), a methylation-resistant cytosine analog, failed to induce *Smad3* expression despite significantly increasing *Rarb* expression, a RA target gene that is also regulated through a CpG island [35]. A 24-h treatment with AZA, however, failed to induce *Smad3*, suggesting that methylation of the CpG island is not the primary mode of regulation for *Smad3* expression (Additional file 2: Figure S2 A,B). Published ChIP-seq data in which RAR occupancy of DNA elements in embryonic stem cells were mapped [GSM482750] confirmed that RARs do not occupy the promoter region of *Smad3* in mice, but rather appear to occupy a site in the intron between exons 3 and 4 of the gene (Figure 1C). ChIP analysis of RAR occupancy of the putative intronic RARE revealed a significant 4-fold enrichment when compared to IgG controls in C2C12 myoblasts (Figure 1D), suggesting that occupancy of this site by RARs may play a role in the regulation of Smad3 expression by RA.

### Treatment with retinoic acid rescues TGFβ-induced inhibition of myogenesis

**Figure 2:**
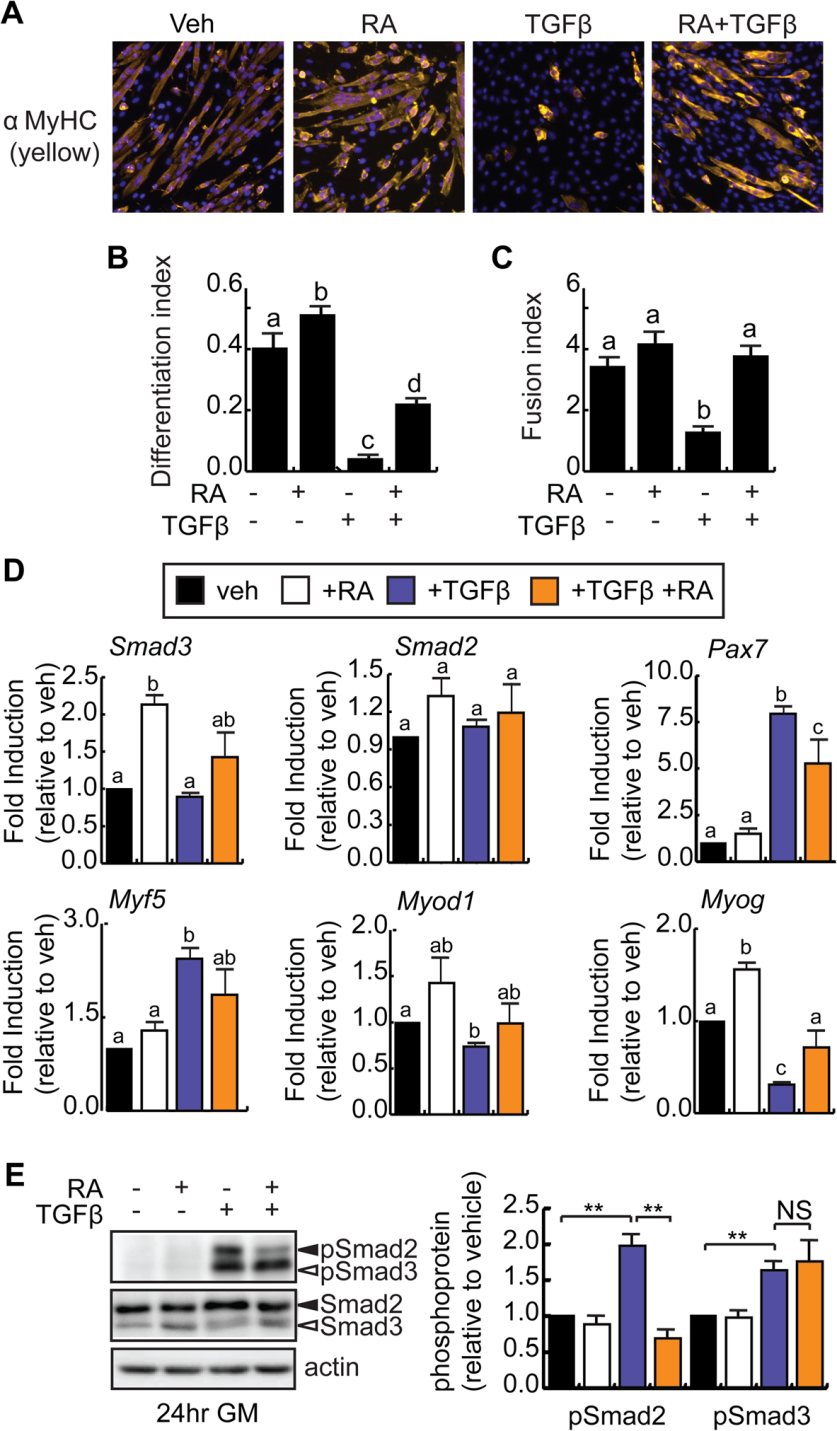
**Retinoic acid partially rescues the inhibition of myogenesis induced by TGFβ. (A)** Representative images of C2C12 myoblasts differentiated in low serum conditions in the continual presence of RA or TGFβ as indicated or with vehicle (Veh) for 96 h and immunostained for myosin heavy chain (MyHC) expression. DAPI is used to reveal nuclei (blue). **(B)** Differentiation index (#myonuclei/#total nuclei) of C2C12 cells cultured and treated as in (A). Means indicated with the same letter are not statistically different from one another. Means with different letters are significantly different from one another with a *P* < 0.05, *n* = 3. **(C)** Fusion index (#myonuclei/#myotubes) of C2C12 cells cultured and treated as in (A). Calculations include mononucleated myosin heavy chain positive cells. Means with different letters are significantly different from one another with a *P* < 0.05, *n* = 3. **(D)** RT-qPCR analysis of *Smad3*, *Smad2*, *Pax7*, and myogenic marker expression in C2C12 myoblasts treated and differentiated as in (A). Means with different letters are significantly different from one another with a *P* < 0.05, *n* = 3. **(E)** Western analysis of phosphorylated Smad2/3 (pSmad2/3) expression in C2C12 cells in growth conditions. Quantification by densitometry is represented as pSmad2 or pSmad3 relative to actin, respectively, *n* = 3, **P < 0.01, NS = not significant. Protein levels for Smad2 and Smad3 are shown.

TGFβ is a well-known reversible inhibitor of myogenesis [12-16,19]. In our experiments, when C2C12 myoblasts were treated with 1 ng/ml TGFβ, cell differentiation in low serum conditions was inhibited, with only a few small myosin heavy chain-expressing cells observed (Figure 2A). The differentiation index (#myonuclei/#total nuclei) of TGFβ-treated cells was reduced 90%, while the fusion index (#myonuclei/#myotubes) was reduced 63%, as compared to vehicle-treated cells (Figure 2B,C). In contrast, treatment with RA alone resulted in a modest but significant increase in differentiation, as compared to vehicle-treated cells (Figure 2A,B) [36-39]. Fusion was unaffected by treatment with RA (Figure 2C). When C2C12 myoblasts were treated with both RA and TGFβ, the differentiation index was partially restored to 55% of vehicle-treated cells (Figure 2A,B), while the fusion index was restored to vehicle-treated levels (Figure 2C).

To better understand the blockade of differentiation in TGFβ-treated cells, RT-qPCR analysis of mRNA expression in C2C12 myoblasts after 96 h in DM was performed (Figure 2D). RA treatment resulted in a significant increase in *Smad3* and *Myog* expression, but did not affect the expression of *Smad2*, *Pax7*, *Myf5*, or *Myod1* as compared to vehicle-treated controls (Figure 2D). By contrast, treatment with TGFβ significantly increased *Pax7* and *Myf5* expression, while decreasing *Myod1* and *Myog* expression (Figure 2D), suggesting a blockade in differentiation in these cells. *Smad3* and *Smad2* mRNA expressions were not affected by TGFβ treatment at this time point (Figure 2D). Co-treatment with both TGFβ and RA significantly reduced *Pax7* expression as compared to TGFβ treatment alone and restored *Myog* expression to the level of vehicle-treated controls, without affecting *Myf5* or *Myod1* levels (Figure 2D). Taken together, this data suggests that RA treatment can partially reverse the inhibitory effects of TGFβ on myoblasts.

Since the inhibition of C/EBPβ activities by Smad3 in preadipocytes and osteoblasts was mediated by Smad3 that was not phosphorylated on its C-terminal tail, as occurs with TGFβ treatment, we examined the classical Smad2/3 C-terminal serine phosphorylation sites in C2C12 myoblasts following a 24-h treatment with TGFβ and RA (Figure 2E). Western analysis to detect phospho-Smad2/3 revealed that while treatment with RA did not induce C-terminal phosphorylation of either Smad, treatment with TGFβ in the presence or absence of RA resulted in robust phosphorylation of the Smad3 S423 and S425 residues (Figure 2E). These results suggest that the effects of RA on TGFβ-mediated signaling are not due to changes in Smad3 phosphorylation status. However, in cells treated with both TGFβ and RA, we did detect a significant decrease in Smad2 phosphorylation of the equivalent residues without changes in Smad2 levels (Figure 2E), suggesting that RA may interfere with the actions of TGFβ by reducing Smad2 activation by phosphorylation.

### TGFβ treatment of myoblasts stimulates C/EBPβ expression

**Figure 3:**
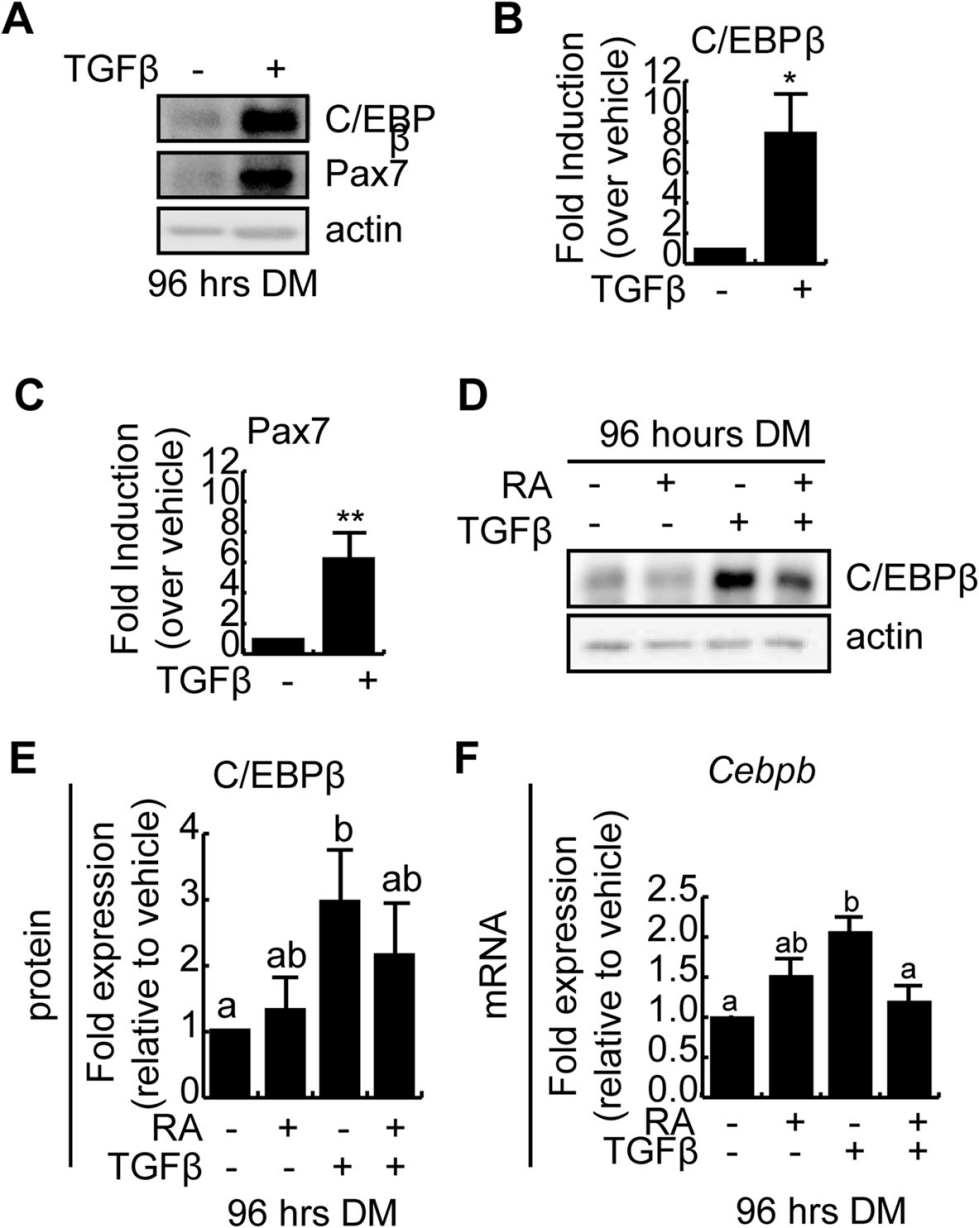
**TGFβ treatment stimulates C/EBPβ expression in myoblasts. (A)** Western analysis of C/EBPβ and Pax7 expression in C2C12 myoblasts cultured in differentiation medium in the presence or absence of TGFβ for 96 h. Actin is used as a loading control. **(B)** Quantification of C/EBPβ expression from western blots in panel (A) relative to vehicle-treated controls. Error bars are the SEM, *n* = 3, *P < 0.05. **(C)** Quantification of Pax7 expression from western blots from (A) relative to vehicle-treated controls. Error bars are the SEM, *n* = 3, **P < 0.01. **(D)** C/EBPβ protein expression after 96 h in differentiation medium (DM) in the continual presence of TGFβ and/or RA, as indicated. Actin is used as a loading control. **(E)** Quantification of western blots from (D) relative to the vehicle-treated controls. Error bars represent the SEM. Means marked with different letters are statistically different, meeting a minimum cutoff of *P* < 0.05, *n* = 4. **(F)** *Cebpb* mRNA expression in C2C12 myoblasts induced to differentiate in the presence or absence of TGFβ and RA for 96 h. Data is shown relative to vehicle-treated controls, and error bars are the SEM, *n* = 3. Means marked by different letters are statistically different from one another, meeting the minimum cutoff of *P* < 0.05.

Given that TGFβ treatment could enhance *Pax7* expression in myoblasts and that *Pax7* is a C/EBPβ target gene in myoblasts [31], we examined C/EBPβ expression levels in TGFβ and RA-treated cells. A 96-h TGFβ treatment of differentiating C2C12 myoblasts resulted in robust 8-fold increase in C/EBPβ expression (Figure 3A,B). TGFβ treatment also resulted in a 6-fold increase in Pax7 expression (Figure 3A,C). We next evaluated the effect of RA on C/EBPβ expression. While RA treatment did not change C/EBPβ expression, co-treatment with both TGFβ and RA under these conditions trended towards decreased C/EBPβ expression, but this failed to meet statistical significance when compared to TGFβ-treated cells (Figure 3D,E). RT-qPCR analysis revealed that while RA had no effect on *Cebpb* mRNA under differentiation conditions, TGFβ could upregulate *Cebpb* mRNA expression by approximately 2-fold under differentiation conditions, as compared to vehicle treatment, and this was prevented with TGFβ and RA co-treatment (Figure 3F). Taken together, these results suggest that C/EBPβ expression is regulated by TGFβ signaling and its expression can be inhibited by co-treatment with RA.

### RA interferes with C/EBPβ occupancy of target genes

**Figure 4:**
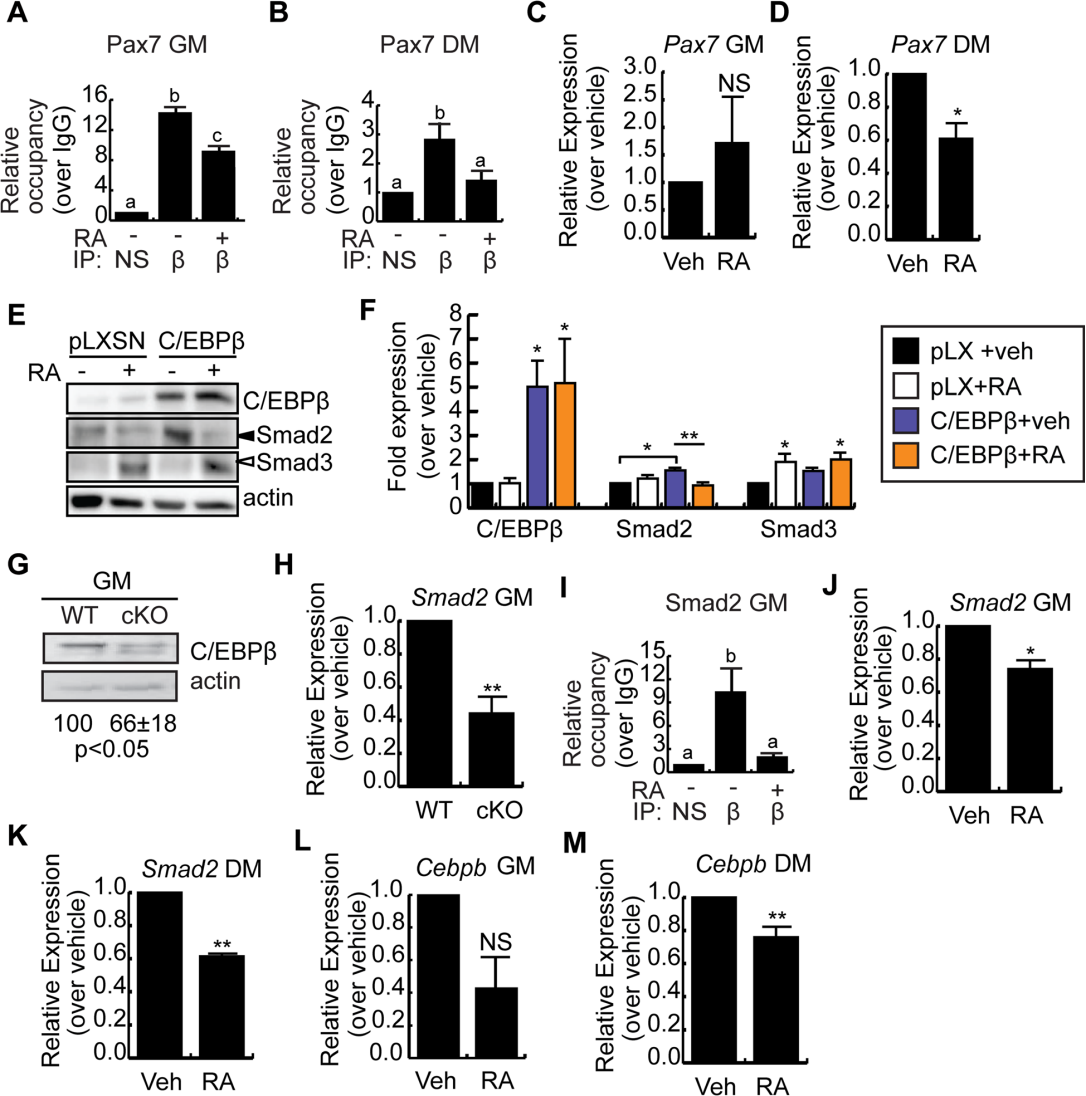
**RA interferes with C/EBPβ occupancy of target genes. (A)** C/EBPβ occupancy of the *Pax7* promoter in C2C12 myoblasts in GM after 24-h +/- RA. *n* = 3. **(B)** C/EBPβ occupancy of the *Pax7* promoter in C2C12 myoblasts after 24-h in DM, +/-RA. *n* = 3. **(C)** *Pax7* expression in C2C12 myoblasts after 24 h +/- RA in GM. *n* = 3. **(D)** *Pax7* expression in C2C12 myoblasts after 24 h +/-RA in DM. *n* = 3. **(E)** Smad2/3 and C/EBPβ expression in C2C12 myoblasts retrovirally transduced to express C/EBPβ or with empty virus (pLXSN) +/- RA for 48 h in GM. Actin is the loading control. **(F)** Quantification of blots from (E); *P < 0.05, compared to vehicle-treated empty virus controls; **P < 0.01, compared to C/EBPβ-overexpressing vehicle-treated cells, *n* ≥ 3. **(G)** C/EBPβ expression in primary myoblasts from C/EBPβ conditional null (cKO) or control (WT) muscle in GM.. Quantification of three trials is indicated, with control set to 100, *P* < 0.05. **(H)** *Smad2* expression in control (WT) and cKO myoblasts in GM. *n* = 3. **(I)** ChIP analysis of C/EBPβ occupancy of the *Smad2* promoter in C2C12 myoblasts after 24-h +/- RA in GM. *n* = 3. **(J)** *Smad2* expression in C2C12 myoblasts treated for 24 h +/-RA in GM. *n* = 3. **(K)** *Smad2* expression in C2C12 myoblasts treated for 24 h +/-RA in DM. *n* = 3. **(L)** *Cebpb* expression in cells cultured as in (J). **(M)** *Cebpb* expression in cells cultured as in (K). *n* = 3. For all graphs, data is the mean +/- SEM. Means marked with different letters are statistically different from one another, meeting a minimum cutoff of *P* < 0.05. *P < 0.05, **P < 0.01. NS = non significant.

One of the consequences of enhanced Smad3 nuclear expression in the absence of robust C-terminal phosphorylation in preadipocytes and mesenchymal stem cells is interference with the transcriptional activities of C/EBPβ [31]. We therefore predicted that RA treatment would decrease C/EBPβ occupancy of key target genes during myogenesis. We have previously demonstrated that *Pax7* is a target gene of C/EBPβ in myoblasts and that C/EBPβ binds to a DNA response element in the *Pax7* promoter [31]. To determine if RA promotes myogenesis by interfering with the activity of C/EBPβ, we performed a chromatin immunoprecipitation (ChIP) assay to evaluate C/EBPβ occupancy of the *Pax7* promoter in C2C12 myoblasts. Since induction to differentiate rapidly reduces Pax7 expression, we treated C2C12 myoblasts for 24 h with RA in growth medium and analyzed C/EBPβ occupancy (Figure 4A). C/EBPβ was readily detected at the *Pax7* promoter in vehicle-treated cells, and RA treatment significantly reduced the occupancy of C/EBPβ at its response element (Figure 4A). We repeated the ChIP in C2C12 myoblasts induced to differentiate for 24 h in the presence or absence of RA and found that RA treatment also decreased C/EBPβ occupancy of the *Pax7* promoter as compared to vehicle-treated cells (Figure 4B).

To correlate changes in occupancy with changes in gene expression, the expression of *Pax7* was evaluated following a 24-h RA treatment under growth conditions (Figure 4C). *Pax7* expression was unaffected by RA treatment in GM, a condition where C/EBPβ promoter occupancy was reduced. However, under differentiation conditions, RA treatment significantly reduced the expression of *Pax7* (Figure 4D), suggesting that the regulation of Pax7 expression in growth medium is less dependent on the actions of C/EBPβ as compared to DM conditions.

Since TGFβ treatment increased C/EBPβ expression (Figure 3A), and persistent expression of C/EBPβ inhibits myogenic differentiation [31], we next examined the regulation of Smad2 and Smad3 in cells overexpressing C/EBPβ. We created pooled stable cell lines by retroviral transduction of C2C12 myoblasts to express C/EBPβ or empty virus control (pLXSN) and then treated the cells with RA for 48 h in growth medium (Figure 4E,F). RA treatment stimulated Smad3 expression in empty virus control cells and C/EBPβ-overexpressing cells (Figure 4E,F). Overexpression of C/EBPβ resulted in a stimulation of Smad2 expression that was reduced when cells were treated with RA (Figure 4E,F), suggesting that RA can interfere with Smad2 expression increased by C/EBPβ. Indeed, in primary myocytes isolated from a conditional null model in which C/EBPβ is excised in Pax7+ cells (Figure 4G), the mRNA expression of *Smad2* was reduced by approximately 60% (Figure 4H). ChIP analysis revealed that C/EBPβ could occupy the *Smad2* promoter in growth conditions (Figure 4I), and this occupancy was reduced to control IP levels by RA treatment, suggesting that RA treatment can also prevent C/EBPβ occupancy of the *Smad2* promoter. The reduction in occupancy was accompanied by a significant decrease in *Smad2* mRNA expression in RA-treated cells in both growth medium and under differentiation conditions (Figure 4J,K), indicating that *Smad2* is a C/EBPβ target gene in this system, and raising the possibility that RA-upregulated Smad3 could prevent Smad2 upregulation and thereby promote myogenesis.

C/EBPβ can also autoregulate its expression [40-42]. Following a 24-h RA treatment under growth conditions, *Cebpb* expression was reduced, though not significantly (*p* < 0.06) (Figure 4L). However, under differentiation conditions, RA treatment significantly reduced the expression of *Cebpb* (Figure 4M), suggesting that RA treatment can decrease the expression of genes that inhibit myogenesis.

### Inhibition of myogenesis by TGFβ is partially rescued by loss of C/EBPβ in myoblasts

**Figure 5:**
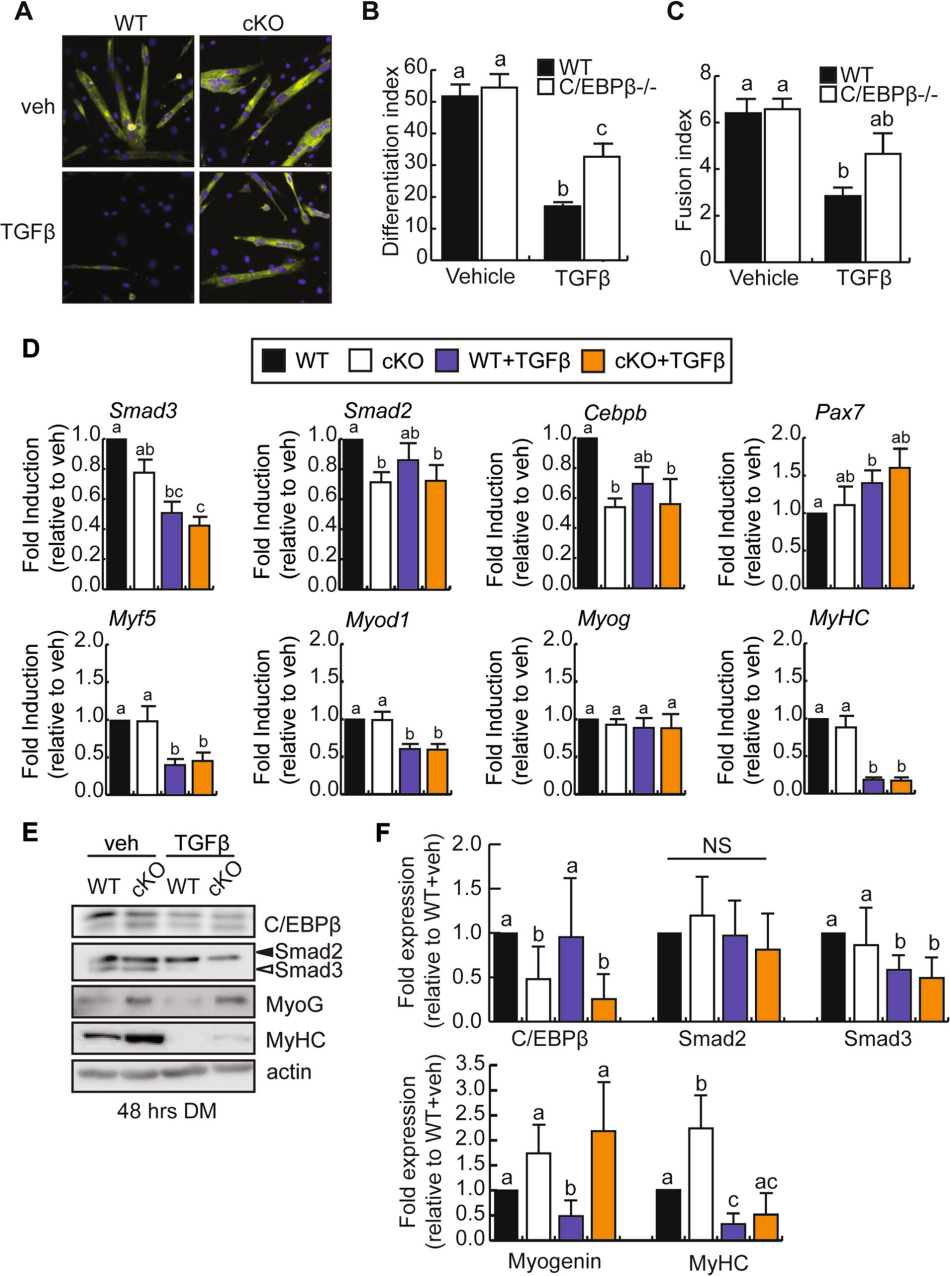
**Inhibition of myogenesis by TGFβ is partially rescued by loss of C/EBPβ expression. (A)** Representative images of myosin heavy chain expression by immunocytochemistry in primary myoblasts isolated from cKO or control (WT) mouse hindlimb and induced to differentiate in low serum for 48 h in the absence (veh) or presence of TGFβ treatment. **(B)** Differentiation indices (#myonuclei/#total nuclei) of cells cultured and treated as in (A). Means marked by different letters are statistically different from one another, with a minimum of *P* < 0.05, *n* = 3. Error bars represent the SEM **(C)** Fusion indices (#myonuclei/#myotubes) of cells cultured and treated as in (A). Counts exclude mononucleated myosin heavy chain positive cells. Error bars represent the SEM; means marked by different letters are statistically different from one another, meeting a minimum cutoff of *P* < 0.05, *n* = 3. **(D)** RT-qPCR analysis of *Smad2*, *Smad3*, *Cebpb*, *Pax7*, and myogenic marker expression in primary myoblasts differentiated as in (A). Means with different letters are significantly different from one another with a *P* < 0.05, *n* ≥ 3. **(E)** Representative western blots of C/EBPβ, Smad2, Smad3, myogenin, and myosin heavy chain in primary myoblasts differentiated as in (A). Means with different letters are significantly different from one another with a *P* < 0.05, *n* = 5. **(F)** Quantification of C/EBPβ, Smad2, Smad3, myogenin, and myosin heavy chain protein expression from (E) relative to vehicle-treated WT control cells. Error bars are the SEM. Means marked with different letters are statistically different from one another, meeting a minimum cutoff of *P* < 0.05, *n* ≥ 3.

Given that C/EBPβ expression is enhanced by TGFβ treatment, we hypothesized that TGFβ inhibits myogenic differentiation at least in part through the induction of C/EBPβ expression. We isolated primary myoblasts from a conditional knockout model (cKO; Cebpb^fl/fl^/Pax7^CreER/+^) and from control mice (WT; Cebpb^fl/fl^/Pax7^+/+^) and induced excision of *Cebpb* in cells expressing CreER with 4-OH tamoxifen. Primary myoblasts differentiate more rapidly than C2C12 myoblasts when exposed to low serum conditions, requiring a shorter time point for evaluation of differentiation. Myogenic differentiation was quantified using immunocytochemistry directed at myosin heavy chain in both WT and cKO cultures (Figure 5A). Vehicle-treated cKO myoblasts differentiated and fused similarly to WT controls (Figure 5A–C). The low dose of TGFβ used (5 ng/ml), however, decreased the number of myosin heavy chain positive cells in WT primary cells, decreasing the differentiation index by 67% as compared to vehicle-treated controls (Figure 5B). Of the TGFβ-treated WT cells that did differentiate, their fusion was severely compromised, with a fusion index (excluding mononuclear cells) reduced by 45% as compared to vehicle-treated controls (Figure 5C). The cKO primary myoblasts were less sensitive to the effects of TGFβ, with a differentiation index significantly higher than WT TGFβ-treated cells, but still distinct from controls (37% decrease as compared to vehicle-treated WT cells) (Figure 5B). The fusion of cKO cells treated with TGFβ, while trending towards restoration, was not statistically different from TGFβ-treated WT cells or vehicle-treated controls (Figure 5C), indicating a partial restoration of myogenesis with loss of C/EBPβ. Indeed, we have observed previously that the conditional loss of C/EBPβ can enhance myoblast fusion [31].

RT-qPCR analysis of myogenic gene expression was performed 48 h after induction to differentiate in the presence or absence of TGFβ in both WT and cKO cells (Figure 5D). TGFβ treatment decreased *Smad3* expression, and this was not dependent on *Cebpb* expression. *Smad2* expression was decreased with loss of C/EBPβ but was not affected by TGFβ treatment. *Pax7* expression was increased by TGFβ treatment in WT cells, but was unaffected by loss of *Cebpb* expression. Both *Myf5* and *Myod1* were decreased by TGFβ treatment of WT cells, and this was not rescued by concomitant loss of *Cebpb*. Surprisingly, despite a partial rescue of the differentiation index, *Myog* expression was unchanged by TGFβ treatment and was unchanged in the cKO cells. Further, *MyHC* expression was, however, decreased by TGFβ treatment in cells of both genotypes. However, western analysis revealed that while the trends we observe at the mRNA level are preserved in protein expression for C/EBPβ and Smad3, myogenin protein expression is restored to control levels in TGFβ-treated cKO cells following differentiation, consistent with our differentiation assay results (Figure 5E,F). Further, myosin heavy chain expression was also increased in the TGFβ-treated cKOs as compared to the WTs though not restored to vehicle-treated levels, suggesting only a partial rescue of differentiation, which can be accounted for by the incomplete knockdown of C/EBPβ in the cKO cells. Smad2 protein levels were also not significantly affected by TGFβ treatment or loss of C/EBPβ expression at this time point, in contrast to changes in mRNA expression.

Taken together, these results suggest that TGFβ acts in part to inhibit myogenic differentiation through the actions of C/EBPβ, either through stimulation of its expression as observed in C2C12 myoblasts or by decreasing Smad3 expression, which is known to interact with and inhibit the DNA occupancy of C/EBPβ.

## Discussion

**Figure 6:**
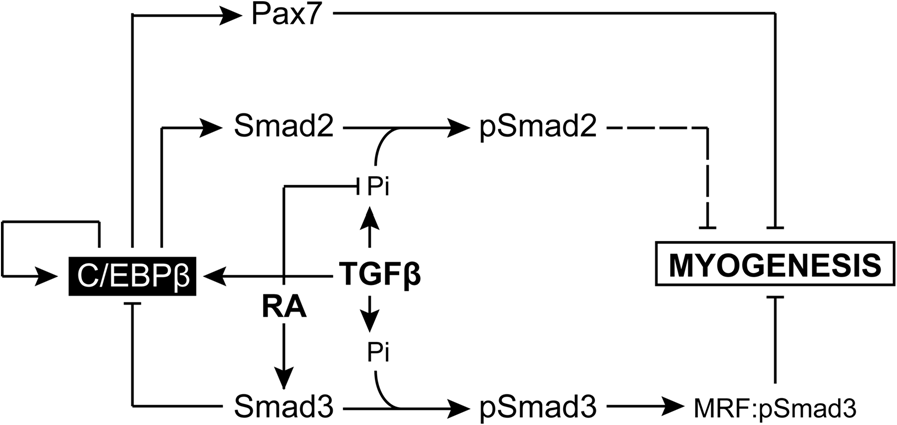
**Proposed pro-myogenic role for retinoic acid and Smad3.** The bzip transcription factor C/EBPβ is a potent inhibitor of myogenesis. It can stimulate the expression of Pax7 and Smad2 and can autoregulate its own expression. Regulation of Pax7 expression suppresses the myogenic differentiation of myoblasts, while the contribution of Smad2 remains unknown. TGFβ treatment can stimulate C/EBPβ expression during myogenic differentiation, and this contributes in part to the anti-myogenic effects of TGFβ treatment as loss of C/EBPβ partially restores differentiation in the presence of TGFβ. TGFβ can also, upon binding its receptor, stimulate the phosphorylation of both Smad2 and Smad3, and it is known that Smad3 can form inhibitory complexes with myogenic regulatory factors and inhibit myogenesis. Treatment with retinoic acid (RA) can antagonize the effects of TGFβ during myogenesis. In particular, RA can stimulate the expression of Smad3 which in turn associates with C/EBPβ and reduces its occupancy of target gene promoters, resulting in a reduction of their expression and the restoration of differentiation. RA treatment can also prevent the normal phosphorylation of Smad2 by the TGFβ receptor, without affecting Smad3 phosphorylation.

Our results place the bzip transcription factor C/EBPβ as a mediator of TGFβ signaling in myoblasts (Figure 6). TGFβ treatment of C2C12 myoblasts stimulates C/EBPβ expression which in turn stimulates the expression of Pax7 and Smad2 as well as autoregulating itself in both growth and differentiation conditions. Indeed, C/EBPβ is known to regulate its own expression through binding to C/EBP elements in its promoter [40-42]. TGFβ treatment also decreased Smad3 expression, as was observed in human osteoarthritic chondrocytes, but this was not dependent on C/EBPβ [26]. Treatment with RA increased Smad3 levels in both growth and differentiation conditions, and decreased C/EBPβ occupancy of its target genes *Pax7* and *Smad2*, resulting in a decrease in the expression of these factors and the stimulation of myogenesis. RA treatment also reduced the phosphorylation of Smad2 in TGFβ-treated cells without affecting Smad3 phosphorylation suggesting a second possible mechanism by which RA can promote myogenesis.

Despite robust upregulation of C/EBPβ expression in TGFβ-treated C2C12 myoblasts, and a role for C/EBPβ in the maintenance of *Smad2* mRNA expression under growth conditions in primary myoblasts, we failed to observe a consistent upregulation of *Smad2* or *Cebpb* expression by TGFβ in the primary myoblasts, while *Pax7* was upregulated. Despite this, in primary myoblasts in which C/EBPβ expression was knocked down, differentiation was partially restored, suggesting that C/EBPβ is an important mediator of the anti-myogenic effects of TGFβ. In the absence of TGFβ treatment and in primary myoblasts, Smad2 expression is readily detectable and could transmit the effects of this signaling pathway independent of C/EBPβ. Indeed, the stimulation of C/EBPβ expression by TGFβ may serve to amplify the response to TGFβ, rather than to mediate it entirely. However, the partial rescue of differentiation suggests that TGFβ also exerts anti-myogenic effects via other mechanisms. While RA treatment restored fusion to control levels, loss of C/EBPβ alone was unable to do so, despite only partial restoration of differentiation in both models. These results suggest that TGFβ can act independently of C/EBPβ to inhibit myoblast fusion. Interestingly, of the proteins known to be implicated in the fusion of myoblasts, specifically ICAM-1, myomaker, Cdk16, and IL-4, none have promoter regions bound by C/EBPβ [43-47]. Further, the expression of myomaker and Cdk16, based on Chip-seq data, appears to be targets of MyoD and myogenin, and MyoD is known to be inhibited by activated Smad3, providing a non-C/EBPβ-dependent mechanism for the inhibition of fusion by TGFβ [12,13,48].

The formation of Smad3-containing inhibitory complexes with MEF2 and MyoD requires C-terminal phosphorylation of serines 423/425 of Smad3 by the liganded TGFβ receptor [12,13]. In RA-treated myoblasts, where Smad3 levels are increased but Smad3 remains dephosphorylated, myogenesis is potentiated and can antagonize the actions of TGFβ. As such, the phosphorylation of Smad3 by the TGFβ receptor may act as a switch trigger, toggling between the pro-myogenic actions of Smad3 in the absence of serine 423/425 phosphorylation and the anti-myogenic state when C-terminally phosphorylated. Indeed, RA treatment had no impact on Smad3 serine 423/425 phosphorylation in our experiments (Figure 2E). This model is consistent with the observation that loss of Smad3 in mice produced smaller muscle fiber diameter, impaired muscle regeneration following acute injury, and reduced satellite cell activation [20,21]. Further, ChIP-seq studies in embryonic stem cells and myoblasts have shown that upon TGFβ treatment, Smad3 co-occupies cell-specific DNA sites with master transcription factors Oct4 and MyoD, respectively, an activity that, in myoblasts, stands paradoxically in opposition to the biological impact of TGFβ in these cells [49]. However, if Smad3 has a pro-myogenic role, it could help prime cells for efficient differentiation by placing MyoD on target promoters. Further, a pro-myogenic role for Smad3 is consistent with our own studies which place RA-induced Smad3 as an important inhibitor of C/EBPβ transcriptional activities in both preadipocytes and mesenchymal stem cells, and an inhibitor of MyoD expression and activity in myoblasts [11,27,29,31].

Despite the absence of serine 423/425 phosphorylation in RA-treated myoblasts, it is possible that Smad3, in these cells, is alternatively phosphorylated or otherwise post-translationally modified following RA treatment. Indeed, numerous phosphorylation sites have been identified in the Smad3 linker region which can have an impact on Smad3 nuclear-cytoplasmic localization, stability and activity [50-54].

Therefore, it remains possible that in unspecified stem cells and in early myogenesis, when C/EBPβ levels are high and consequently MyoD levels are low, Smad3 expression can act to promote myogenesis at least in part through the inhibition of C/EBPβ activity (Figure 6). However, once C/EBPβ levels are downregulated, as they are in C2C12 myoblasts, and MyoD expression increases, Smad3 could become available to interfere with MyoD and MEF2-containing transcriptional complexes and exert an anti-myogenic role. As such, the inhibition of myogenesis by TGFβ may occur through at least two distinct pathways, one mediated by C/EBPβ and a second occurring downstream of myogenic regulatory factor activation.

## Conclusions

We provide evidence that C/EBPβ is a non-canonical regulator of TGFβ signaling. TGFβ signaling stimulates C/EBPβ expression, which in turn increases Smad2 expression and Pax7 expression in myoblasts, inhibiting differentiation. Loss of C/EBPβ expression, or interference with C/EBPβ activity via RA treatment in myoblasts, resulted in a partial rescue of differentiation in the presence of TGFβ, suggesting that TGFβ works at least in part through C/EBPβ to inhibit myogenesis.

## Additional files

Additional file 1: Figure S1. High doses of retinoic acid reduces C2C12 cell number. **(A)** C2C12 myoblasts were induced to differentiate in low serum conditions for 96 h in the absence or presence of all-*trans* retinoic acid (RA) at indicated doses. Cells were then fixed and subjected to immunostaining for myosin heavy chain expression and counterstained with DAPI to reveal nuclei. Representative images are shown. **(B)** DAPI cell counts per field of view at increasing doses of RA in C2C12 cells differentiated as in (A) for 96 h. Error bars are the SEM, **P* < 0.05, NS = not significant, *n* = 5. **(C)** Crystal violet assay measuring C2C12 cell number after 1, 2, or 3 days in growth medium in the presence or absence of 1 nM RA, *n* = 3; error bars are the SEM. Differences are not statistically significant. Additional file 2: Figure S2. Induction of Smad3 expression by RA is direct. **(A)** Quantitative PCR analysis of *Smad3* mRNA expression following treatment of 3T3-L1 cells with RA or vehicle for 8 h and azacytidine (AZA) or vehicle for 24 h. 5-Azacytadine (Sigma-Aldrich) was used at a concentration of 3 μM. Data is represented as fold expression over vehicle-treated. Error bars are the SEM. Means marked with different letters are statistically different with a minimum threshold of *P* < 0.05, *n* = 3. **(B)** Quantitative PCR analysis of *Rarb2* mRNA following treatment of 3T3-L1 cells with RA or vehicle for 8 h and azacytidine or vehicle for 24 h. Data is represented as fold expression over vehicle-treated. Error bars are the SEM. Means marked with different letters are statistically different with a minimum cutoff of *P* < 0.05, *n* = 3.

## Abbreviations

C/EBPβ: CCAAT/enhancer binding protein beta
RA: Retinoic acid
TGFβ: Transforming growth factor-beta
GM: Growth medium
DM: Differentiation medium
RARE: Retinoic acid response element
RAR: Retinoic acid receptor
