# Supplementary figures and images for: Retinoic acid promotes myogenesis in myoblasts by antagonizing transforming growth factor-beta signaling via C/EBPβ
Source: Skelet Muscle. 2015 Mar 18;5:8. doi: 10.1186/s13395-015-0032-z (PMC4397812; doi:10.1186/s13395-015-0032-z)

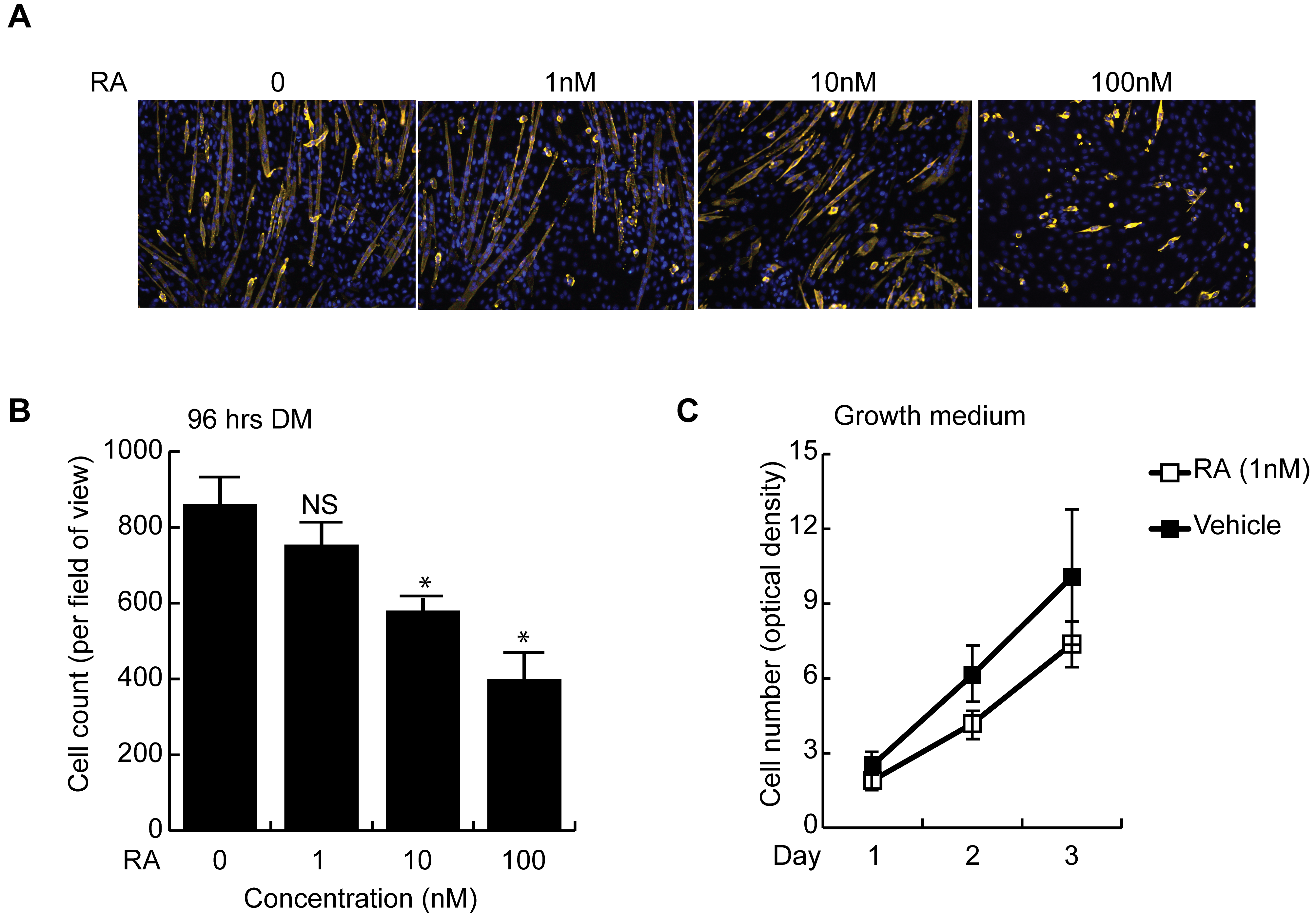

Supplement: Additional file 1: Figure S1. — High doses of retinoic acid reduces C2C12 cell number. (A) C2C12 myoblasts were induced to differentiate in low serum conditions for 96 h in the absence or presence of all-trans retinoic acid (RA) at indicated doses. Cells were then fixed and subjected to immunostaining for myosin heavy chain expression and counterstained with DAPI to reveal nuclei. Representative images are shown. (B) DAPI cell counts per field of view at increasing doses of RA in C2C12 cells differentiated as in (A) for 96 h. Error bars are the SEM, *P < 0.05, NS = not significant, n = 5. (C) Crystal violet assay measuring C2C12 cell number after 1, 2, or 3 days in growth medium in the presence or absence of 1 nM RA, n = 3; error bars are the SEM. Differences are not statistically significant. [file 13395_2015_32_MOESM1_ESM.tiff]

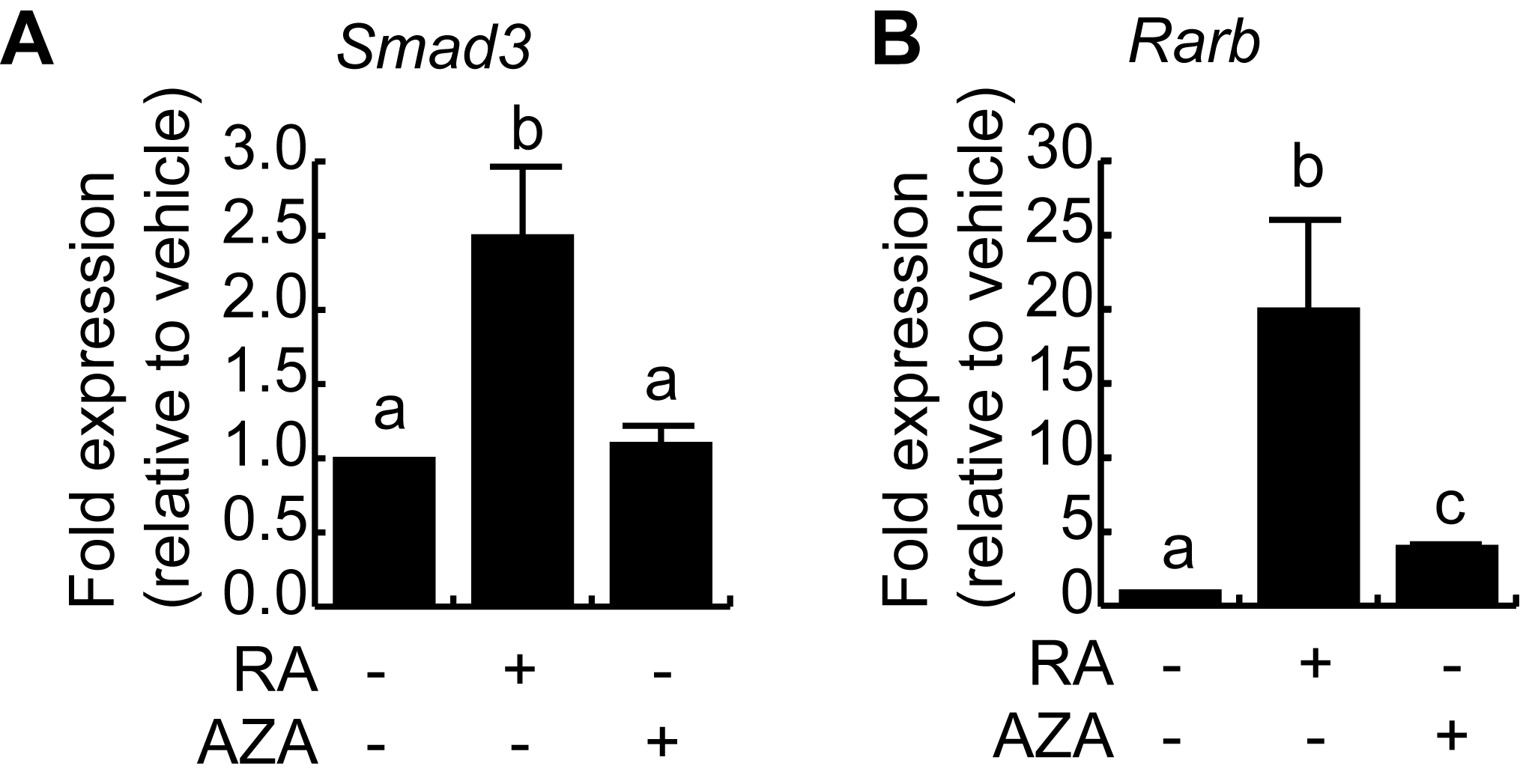

Supplement: Additional file 2: Figure S2. — Induction of Smad3 expression by RA is direct. (A) Quantitative PCR analysis of Smad3 mRNA expression following treatment of 3T3-L1 cells with RA or vehicle for 8 h and azacytidine (AZA) or vehicle for 24 h. 5-Azacytadine (Sigma-Aldrich) was used at a concentration of 3 μM. Data is represented as fold expression over vehicle-treated. Error bars are the SEM. Means marked with different letters are statistically different with a minimum threshold of P < 0.05, n = 3. (B) Quantitative PCR analysis of Rarb2 mRNA following treatment of 3T3-L1 cells with RA or vehicle for 8 h and azacytidine or vehicle for 24 h. Data is represented as fold expression over vehicle-treated. Error bars are the SEM. Means marked with different letters are statistically different with a minimum cutoff of P < 0.05, n = 3. [file 13395_2015_32_MOESM2_ESM.tiff]
